# Reprogramming the tumor microenvironment to overcome immunotherapy resistance in pancreatic cancer

**DOI:** 10.3389/fimmu.2025.1717062

**Published:** 2025-11-18

**Authors:** Xianfeng Hui, Xiaowei Tian, Shihuan Ding, Aiping Sun, Tiesuo Zhao, Hui Wang

**Affiliations:** 1 Department of Immunology, School of Basic Medical Sciences, Henan Medical University, Xinxiang, China; 2 Henan Collaborative Innovation Center of Molecular Diagnosis and Laboratory Medicine, School of Medical Technology, Henan Medical University, Xinxiang, China; 3 Xinxiang Engineering Technology Research Center of Immune Checkpoint Drug for Liver-Intestinal Tumors, Henan Medical University, Xinxiang, China; 4 Department of Pathogenic Biology, School of Basic Medical Sciences, Henan Medical University, Xinxiang, China

**Keywords:** pancreatic ductal adenocarcinoma, tumor microenvironment, immunotherapy resistance, immune evasion, metabolic reprogramming, CAF targeting

## Abstract

**Background:**

Pancreatic ductal adenocarcinoma (PDAC) exhibits profound resistance to immunotherapy due to its highly immunosuppressive tumor microenvironment (TME).

**Objective:**

This review aims to elucidate the key mechanisms of TME-mediated immune evasion in PDAC and explore therapeutic strategies to overcome these barriers.

**Methods:**

A comprehensive analysis of recent studies was conducted, focusing on the cellular, stromal, and metabolic components of the PDAC TME, alongside emerging technologies for TME reprogramming.

**Results:**

Dense extracellular matrix, CAF-driven fibrosis, myeloid-derived suppressor cells (MDSCs), tumor-associated macrophages (TAMs), Tregs, and metabolic competition collectively impair immune cell infiltration and activation. Novel interventions—including ECM remodeling, CAF modulation, metabolic reprogramming, and myeloid cell targeting—show promise in restoring immune responsiveness.

**Conclusion:**

TME reprogramming represents a critical strategy to enhance immunotherapy efficacy in PDAC, offering new opportunities for overcoming immune exclusion and resistance.

## 1 Introduction

Despite the groundbreaking success of immune checkpoint blockade (ICB) therapies in various solid tumors, their efficacy remains severely limited in a subset of immunologically “cold” tumors (1–3). An increasing body of evidence indicates that the tumor microenvironment (TME) constitutes a major barrier to effective immunotherapy, playing a central role in immune resistance and tumor immune evasion (4–6). The TME is composed not only of malignant tumor cells but also a wide array of non-malignant components, including cancer-associated fibroblasts (CAFs), immunosuppressive myeloid cells—such as tumor-associated macrophages (TAMs) and myeloid-derived suppressor cells (MDSCs)—regulatory T cells (Tregs), abnormal vasculature, extracellular matrix (ECM), and a distinct metabolic milieu (7, 8). These elements synergistically establish a profoundly immunosuppressive landscape that hinders immune cell infiltration, activation, and antitumor functionality (9).

Among all malignancies that heavily rely on immune evasion within the TME, pancreatic ductal adenocarcinoma (PDAC) stands out as one of the most representative and challenging models (10). PDAC is characterized by an exceptionally high mortality rate and a dismally low five-year survival rate—less than 9%. It is widely recognized as an “immune desert” tumor, notoriously unresponsive to immunotherapy (11).

A major contributor to this poor prognosis is the difficulty of early detection. Most PDAC cases are diagnosed at an advanced or metastatic stage, largely due to the lack of specific symptoms and reliable biomarkers during early tumor development (12, 13). Recent advances in multi-omics profiling, liquid biopsy, and artificial intelligence–assisted imaging have shown promise in identifying early molecular signatures and circulating tumor components that could enable earlier diagnosis and intervention (14). However, despite these technological breakthroughs, the translation of such diagnostic strategies into clinical practice remains limited, underscoring the urgent need for effective early detection tools that can complement therapeutic innovation.

The TME of PDAC exhibits a densely fibrotic stroma, primarily orchestrated by activated CAFs, which secrete excessive amounts of collagen and hyaluronic acid (15, 16). This creates a formidable physical barrier that severely impedes the infiltration of immune effector cells. Moreover, the PDAC TME is enriched with immunosuppressive cell populations such as TAMs, myeloid-derived suppressor cells (MDSCs), and Tregs (17). These cells continuously release inhibitory cytokines—including interleukin-10 (IL-10) and transforming growth factor-beta (TGF-β)—as well as immunosuppressive metabolic byproducts, collectively driving the functional exhaustion of CD8^+^ T cells (18, 19). Simultaneously, hypoxia, elevated lactate levels, and an acidic microenvironment further compromise the viability and cytotoxic activity of immune cells, reinforcing immune tolerance and facilitating relentless tumor progression (20, 21).

Conventional ICB strategies have shown limited efficacy in PDAC, as monotherapeutic immune activation is insufficient to overcome the profoundly immunosuppressive TME (22). In contrast, therapeutic approaches targeting the TME have emerged as a promising avenue to overcome the immunotherapy resistance observed in PDAC (11, 23). By strategically modulating key components of the TME—such as inhibiting CAF activation, dismantling the dense ECM, reprogramming the function of myeloid-derived immune cells, correcting aberrant metabolic states, and restoring vascular normalization—it is possible to alleviate both physical and immunological barriers (10, 24). These interventions can facilitate the infiltration and reinvigoration of effector immune cells, thereby enhancing the efficacy of ICB and other immunotherapeutic modalities.

Accordingly, this review centers on the theme of “tumor microenvironment reprogramming,” with the aim of systematically elucidating the pivotal mechanisms by which the TME contributes to immune evasion in pancreatic cancer. We provide an in-depth analysis of the current therapeutic strategies and research advances targeting various components of the TME, and explore the potential of TME-directed combination immunotherapies in overcoming resistance in PDAC and other immunologically cold tumors. Through this comprehensive synthesis, we seek to offer a conceptual framework and translational insights that may guide the development of more effective and durable immunotherapeutic approaches.

## 2 The Immunosuppressive TME in PDAC

Among solid tumors, PDAC exemplifies the archetype of an immunologically “cold” malignancy, defined by a deeply immunosuppressive TME that presents formidable barriers to effective immunotherapy (25). Similar to other “cold” tumors such as glioblastoma and prostate cancer, PDAC exhibits profound immune exclusion and myeloid-driven suppression; however, it is uniquely distinguished by an exceptionally dense desmoplastic stroma and rigid metabolic landscape that further restrict immune infiltration. Glioblastoma is dominated by microglial-mediated immunosuppression and the protective constraints of the blood–brain barrier, whereas prostate cancer demonstrates androgen-driven immune modulation and regional T cell exclusion. The TME in PDAC is composed of a diverse array of immunosuppressive cell populations and is further distinguished by extensive stromal remodeling, metabolic dysregulation, and aberrant activation of cytokine networks (10, 26, 27). Collectively, these elements converge to create a microenvironment that is deeply hostile to antitumor immune responses.

### 2.1 Immunosuppressive cellular constituents within the TME

#### 2.1.1 Cancer-associated fibroblasts

CAFs represent one of the most abundant stromal cell populations within the PDAC tumor microenvironment (28). Single-cell transcriptomic profiling has revealed substantial functional and spatial heterogeneity among CAFs, which can be broadly categorized into three subtypes: myofibroblastic CAFs (myCAFs), inflammatory CAFs (iCAFs), and antigen-presenting CAFs (apCAFs) (29, 30). myCAFs, located adjacent to tumor epithelial cells, express high levels of α-smooth muscle actin (α-SMA) and are primarily responsible for extracellular matrix (ECM) deposition, producing collagen and hyaluronic acid that form a dense desmoplastic stroma (31). This fibrotic barrier restricts immune cell infiltration and contributes to the hypoxic, high-pressure microenvironment typical of PDAC.

In contrast, iCAFs, which reside farther from tumor nests, secrete large quantities of pro-inflammatory mediators, including IL-6, CXCL12, and LIF (32, 33). These cytokines not only promote tumor proliferation and survival but also attract and activate immunosuppressive immune cells such as MDSCs and Tregs, thereby amplifying local immune suppression. apCAFs, characterized by the expression of MHC class II but lacking co-stimulatory molecules CD80 and CD86, fail to properly activate CD4^+^ T cells and instead induce tolerance and exhaustion (34). Across CAF subsets, TGF-β secretion plays a central role in sustaining immunosuppression by inhibiting cytotoxic T cell function, enhancing Treg differentiation, and impairing dendritic cell activation (35). Collectively, CAFs construct both a structural and biochemical niche that enforces immune exclusion and sustains the “cold” phenotype of PDAC.

#### 2.1.2 Tumor-associated macrophages

TAMs constitute another dominant immunosuppressive population in the PDAC TME. They are predominantly polarized toward an M2-like phenotype that facilitates tumor progression (36). M2-TAMs secrete high levels of IL-10, TGF-β, and VEGF, which suppress effector T cell activity while promoting angiogenesis and ECM remodeling (18, 37). This dual role reinforces both the physical and immunological barriers that protect the tumor from immune attack (10). In addition, TAMs express immunosuppressive surface molecules such as PD-L1, CD206, and Arginase-1 (Arg1) (38, 39). PD-L1 engagement with PD-1 on T cells induces exhaustion, while Arg1-mediated arginine depletion limits T cell proliferation and effector function (40). Under hypoxic conditions, hypoxia-inducible factor-1α (HIF-1α) further enhances TAM polarization toward the M2 state and upregulates VEGF, exacerbating immunosuppression and vascular abnormalities (41, 42). Moreover, TAMs coordinate closely with other stromal components, recruiting Tregs and monocytes through chemokines (e.g., CCL2, CCL5) and stimulating CAFs via TGF-β-dependent feedback loops, thereby reinforcing the immunosuppressive ecosystem (43, 44).

#### 2.1.3 Regulatory T cells

Tregs are markedly enriched within PDAC lesions and display an activated phenotype characterized by high FoxP3 and CD25 expression (45, 46). They suppress antitumor immunity through multiple mechanisms, including IL-2 consumption, CTLA-4–mediated competition with effector T cells for co-stimulatory signals, and secretion of IL-10 and TGF-β, which collectively inhibit T cell activation and cytotoxicity (43, 47). Furthermore, Tregs impair dendritic cell maturation and antigen presentation, thereby blunting adaptive immune priming (48, 49). In the nutrient-deprived PDAC TME, Tregs exhibit metabolic plasticity, relying on enhanced fatty acid oxidation and mitochondrial oxidative phosphorylation to sustain their suppressive functions under hypoxic stress (50, 51). Through metabolic and cytokine-mediated crosstalk with TAMs and CAFs, Tregs help maintain a self-reinforcing immunoregulatory network that sustains immune tolerance and therapeutic resistance.

#### 2.1.4 Myeloid-derived suppressor cells

**Figure 1:**
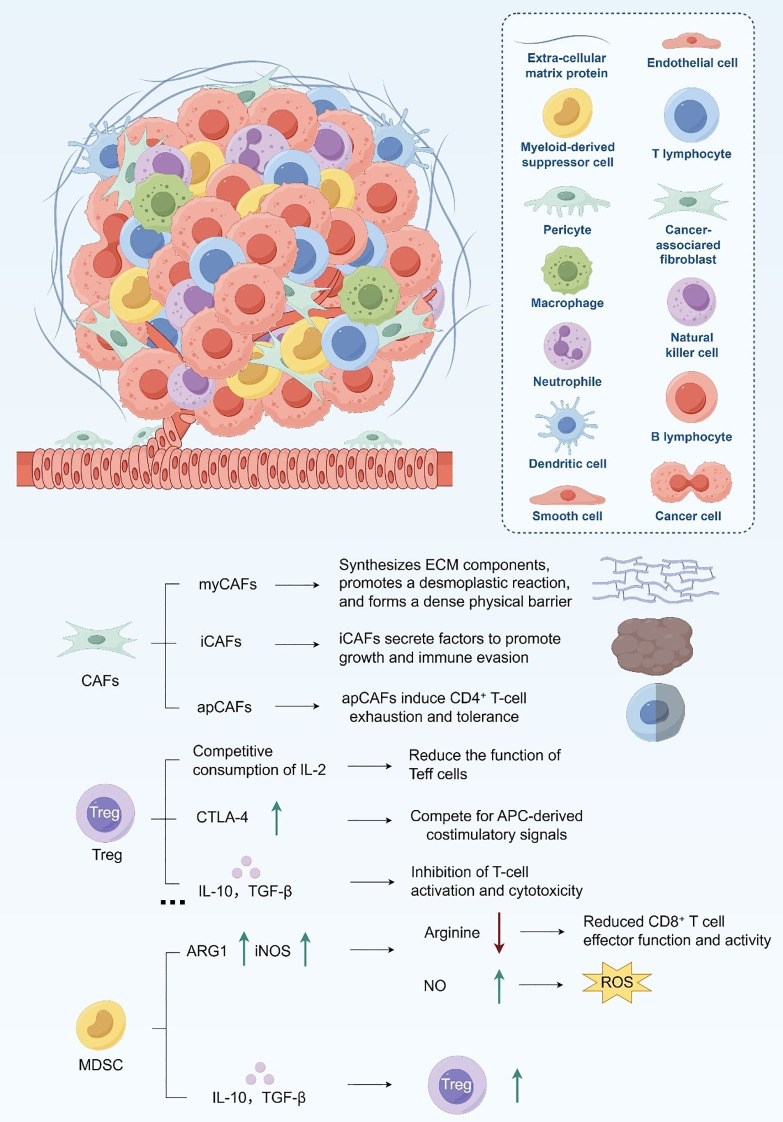
Immunosuppressive cellular constituents within the TME.

MDSCs are highly enriched in PDAC and function as potent inhibitors of antitumor immunity (52, 53). They suppress T cell function through metabolic competition and redox-mediated stress. MDSCs express elevated levels of arginase-1 (ARG1) and inducible nitric oxide synthase (iNOS), which respectively deplete arginine and generate nitric oxide (NO) (54). Arginine depletion limits T cell proliferation, while NO and reactive oxygen species (ROS) disrupt T cell receptor signaling and induce apoptosis (55, 56). In addition, MDSCs sequester cysteine, further impairing T cell redox balance (57). At the immunoregulatory level, MDSCs release IL-10 and TGF-β to promote Treg expansion, and interact with TAMs through reciprocal cytokine loops to amplify immune suppression (58). Together, these mechanisms establish a highly coordinated network that underpins PDAC’s profound resistance to immune-based therapies (**Figure 1**).

### 2.2 Non-cellular barriers and metabolic dysregulation within the TME of PDAC

From a non-cellular perspective, the ECM in PDAC is abnormally abundant and densely structured, constituting a central component of the physical immune barrier (59). The ECM is primarily composed of an extensive network of collagen types I, III, and IV, hyaluronic acid, and fibronectin, forming a highly cross-linked and mechanically rigid matrix that defines the structural landscape of the TME (60). This fibrotic matrix is predominantly secreted and remodeled by activated CAFs, which play a pivotal role in ECM homeostasis (61). CAFs not only synthesize large quantities of ECM components but also regulate their degradation and spatial organization through the secretion of matrix metalloproteinases (MMPs), thereby maintaining elevated matrix tension characteristic of PDAC (62). The excessive deposition of ECM components significantly elevates interstitial pressure, which in turn compresses the tumor vasculature, leading to vascular collapse, impaired perfusion, and the establishment of widespread and chronic hypoxia within the tumor tissue (63, 64). Such hypoxic conditions exert profound immunosuppressive effects by dampening the metabolic activity and functional integrity of immune effector cells. Furthermore, hypoxia promotes the recruitment and polarization of immunosuppressive cell populations—such as MDSCs, TAMs, and Tregs—further reinforcing the immune-refractory state of the TME (65). In addition to its biomechanical role, the ECM actively participates in immunomodulation by engaging with cell surface receptors on immune cells, including integrins and CD44 (66). These interactions initiate a cascade of downstream immunosuppressive signaling pathways, such as focal adhesion kinase (FAK), PI3K-Akt, and TGF-β signaling, which collectively impair T cell trafficking, survival, and cytotoxic function (67).

**Figure 2:**
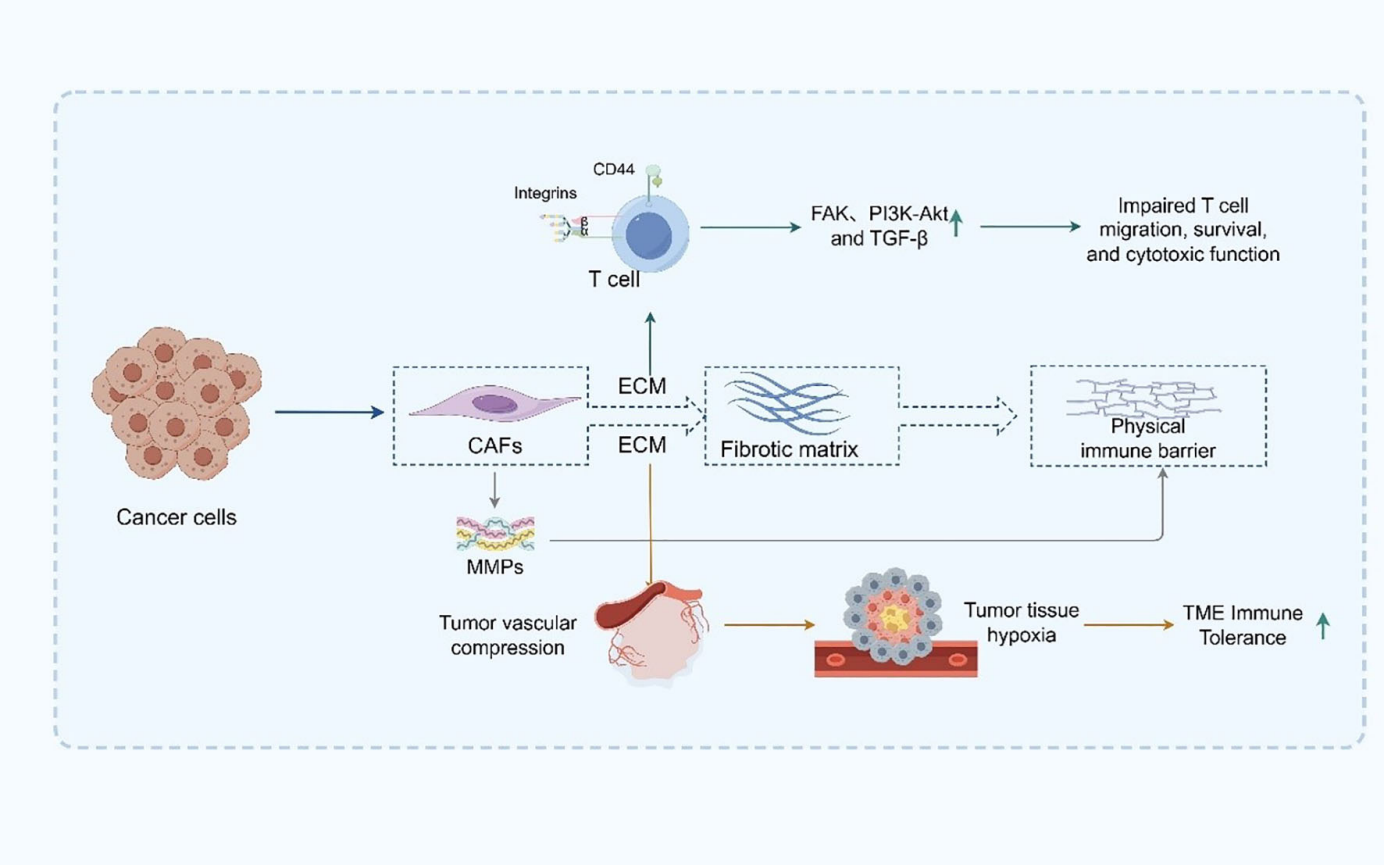
ECM-mediated physical and immunosuppressive barriers in PDAC.

Moreover, the dense ECM architecture impedes the mobility and spatial positioning of DCs and T cells within the tumor, thereby compromising antigen presentation and immune synapse formation (68). This spatial restriction hinders the initiation and execution of effective antitumor immune responses (69) (**Figure 2**). Collectively, the ECM in PDAC is not merely a passive scaffold but rather a dynamic and active regulator of immune suppression. Its abnormal accumulation and remodeling create a dual barrier—both physical and molecular—that shields tumor cells from immune surveillance (70, 71). Targeting ECM components or CAF-mediated matrix remodeling has thus emerged as a promising strategy to decompress the stroma, restore vascular perfusion, enhance immune cell infiltration, and ultimately improve the efficacy of immunotherapy in this notoriously treatment-refractory malignancy.

### 2.3 Hypoxia, metabolic stress, and chemokine signaling as key factors shaping immune suppression in the PDAC tumor microenvironment

Hypoxia and metabolic stress represent another critical axis of immunosuppression within the TME of PDAC (72). Owing to pronounced desmoplastic stroma and continuous deposition of ECM components—such as collagen and hyaluronic acid—by CAFs, the interstitial pressure in PDAC tissue markedly increases (73). This heightened mechanical stress compresses tumor vasculature, resulting in perfusion deficits and the establishment of widespread, persistent hypoxia (74).

Under hypoxic conditions, HIF-1α and HIF-2α are stabilized and initiate a broad transcriptional program that includes the upregulation of VEGF and other pro-angiogenic mediators (75). However, the resulting neovasculature is often structurally aberrant and functionally leaky, further exacerbating local hypoxia and impeding immune cell trafficking. In parallel, HIF signaling also induces the expression of multiple immune checkpoint molecules, including programmed death-ligand 1 (PD-L1) and CD47—the latter serving as a “don’t eat me” signal that suppresses macrophage phagocytosis—thereby directly impairing T cell function and fostering an immunosuppressive milieu (76, 77).

Simultaneously, hypoxia augments aerobic glycolysis (the Warburg effect), leading to significant accumulation of lactate within the TME and the formation of a locally acidic environment (72, 78). This drop in pH directly suppresses the cytotoxic activity of CD8^+^ T cells, promoting their functional exhaustion and impairing proliferative capacity (79). Lactate also acts on tumor-associated macrophages, skewing their polarization toward the M2-like phenotype, which is associated with enhanced immunoregulatory activity and tumor progression (42). In addition, intense metabolic competition between tumor and immune cells for critical nutrients—including glucose, glutamine, and tryptophan—further restricts the metabolic plasticity of T cells, diminishing their capacity to sustain the energetically demanding antitumor response (80).

Beyond hypoxia and metabolic stress, PDAC TME harbors a tightly regulated immunosuppressive signaling network orchestrated by soluble factors, chemokines, and tumor-derived extracellular vesicles (81). CAFs play a central role by secreting CXCL12, which forms a chemokine barrier at the tumor periphery that restricts CD8^+^ T cell infiltration into the tumor core and impairs their spatial positioning (82). CCL2 is abundantly expressed in PDAC and engages the CCR2 receptor on circulating myeloid cells, promoting the recruitment of immunosuppressive MDSCs and TAMs (83). Simultaneously, cytokines such as IL-10 and TGF-β are widely distributed throughout the TME, contributing to the maintenance of immune tolerance by inhibiting DC maturation, impairing antigen presentation, and reinforcing the suppressive function of Tregs (84).

In addition, tumor-derived exosomes have emerged as potent mediators of immune modulation (85, 86). These nanoscale vesicles are enriched in diverse immunoregulatory cargo—including microRNAs (e.g., miR-21, miR-146a), immune checkpoint proteins (e.g., PD-L1, TGF-β), and bioactive lipids—that exert systemic effects on hematopoietic organs (87). By reprogramming myeloid progenitors in the bone marrow, exosomes facilitate the preferential differentiation of MDSCs and other immunosuppressive lineages, thereby reinforcing systemic immune tolerance from its origin.

**Figure 3:**
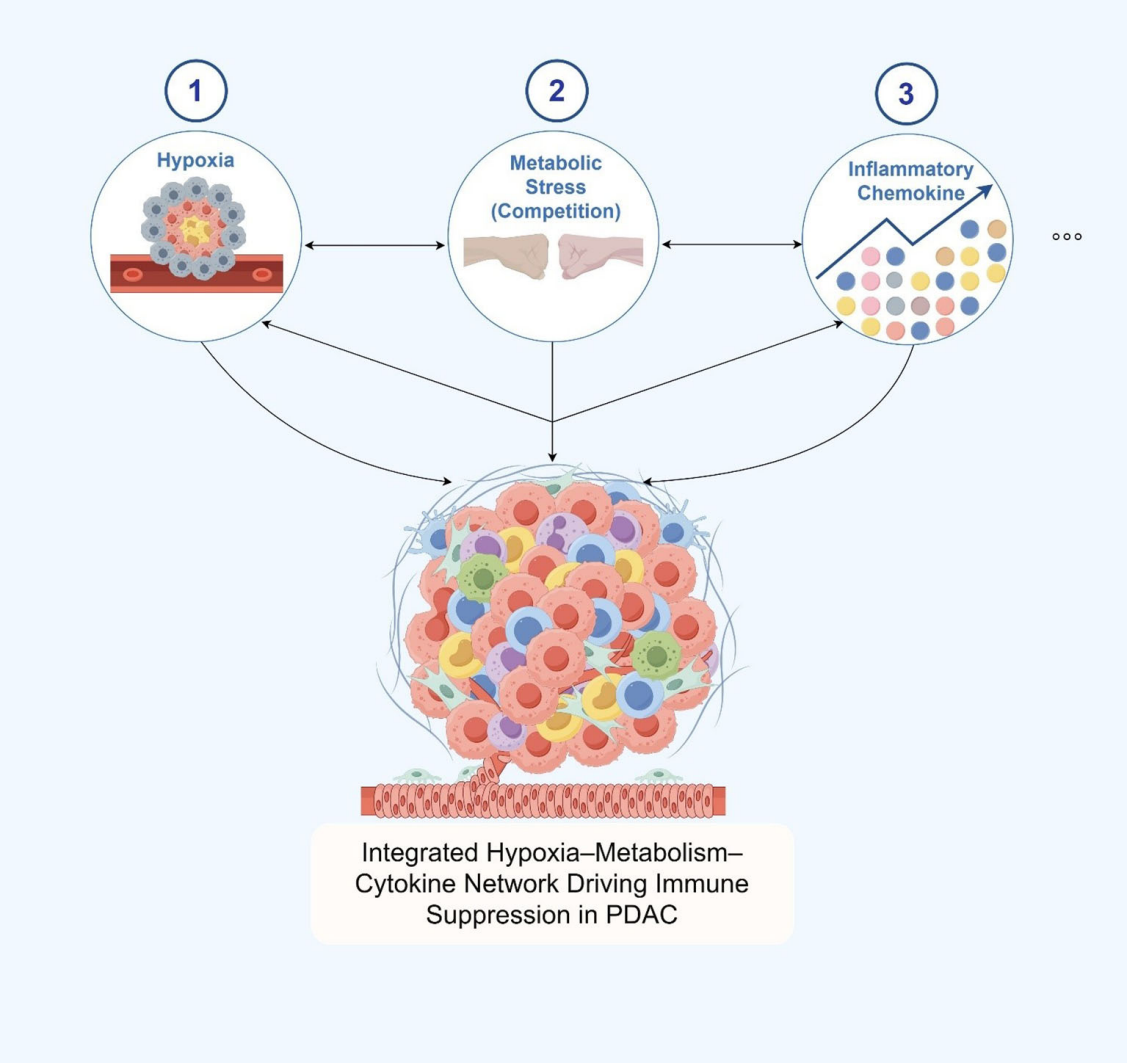
Hypoxia, metabolic stress, and chemokine signaling as key factors shaping immune suppression in the pdac tumor microenvironment.

Collectively, hypoxia, metabolic stress, and inflammatory chemokine signaling coalesce in the PDAC TME to construct a multidimensional and progressively layered immunosuppressive network (**Figure 3**). This network not only constrains the functionality of effector immune cells but also interferes with nutrient availability, spatial immune cell distribution, and long-range immunoregulatory signaling. These integrated mechanisms underlie the profound resistance of PDAC to current immunotherapeutic strategies and have become critical focal points for the development of TME-targeted therapeutic interventions.

## 3 Mechanisms of immune exclusion and resistance to immunotherapy

Building upon the multifaceted cellular, stromal, and metabolic barriers described above, it becomes evident that the immunosuppressive TME of PDAC is not merely a passive consequence of tumor progression but rather an actively organized defense system that enforces immune exclusion and fosters therapeutic resistance. In this context, monotherapy with immune checkpoint blockade has consistently yielded minimal clinical benefit, with objective response rates rarely exceeding 5% (88). Such pronounced refractoriness cannot be attributed to a single molecular lesion but instead reflects a highly orchestrated, multidimensional network of immunological and stromal barriers within the TME (81). Acting in concert, these barriers establish a systemic and resilient architecture of immune evasion and therapeutic resistance, posing a formidable challenge to current immunotherapeutic paradigms (89).

### 3.1 Physical barriers: fibrotic stroma–driven immune exclusion

The uniquely dense fibrotic stroma of PDAC forms one of the most formidable physical barriers to effective antitumor immunity (90). Within the tumor microenvironment (TME), cancer-associated fibroblasts (CAFs) are highly activated and act as the primary source of extracellular matrix (ECM) components, including collagen, hyaluronic acid, and fibronectin (91). The excessive deposition of these ECM elements increases interstitial fluid pressure and compresses intratumoral blood vessels, resulting in poor perfusion and hypoxia. These mechanical and structural alterations create a hostile physical landscape that limits the penetration of effector immune cells—particularly CD8^+^ cytotoxic T lymphocytes and dendritic cells—into the tumor core (92).

In addition to structural impediments, CAFs contribute to spatial immune exclusion through the secretion of chemokines such as CXCL12, which activates the CXCR4 signaling axis and creates a chemotactic barrier that restricts immune cell localization to the tumor periphery. Furthermore, CAFs secrete high levels of TGF-β, which exacerbates ECM deposition while simultaneously exerting potent immunosuppressive effects by inhibiting T cell activation and cytotoxicity (93). The interplay between these mechanical and biochemical signals establishes a dual-layered barrier—both physical and immunological—that represents a primary obstacle to effective immune infiltration and antitumor immunity in PDAC (92).

Beyond the physical barrier, CAFs actively modulate immune exclusion through paracrine signaling. They secrete chemokines such as CXCL12, which activates the CXCR4 signaling axis and spatially confines immune cells to the tumor periphery. In parallel, CAF-derived TGF-β amplifies ECM production while suppressing T cell activation and cytotoxic function (108). Together, these mechanical and biochemical mechanisms form a dual-layered defense system—structural and immunological—that collectively prevents effective immune infiltration and sustains immune evasion in PDAC (107).

### 3.2 Myeloid cell–dominated immunosuppression

Concurrently, the TME of PDAC is heavily infiltrated by immunosuppressive myeloid populations, which collectively establish a profoundly immune-tolerant ecosystem (94). TAMs in PDAC predominantly exhibit an M2-like immunosuppressive phenotype and secrete high levels of IL-10 and TGF-β (95). These factors suppress antigen presentation capacity, upregulate inhibitory molecules such as PD-L1 and arginase-1 (ARG1), and directly impair the cytotoxic function of CD8^+^ T cells (96).

MDSCs contribute to immune evasion through metabolic competition, depleting key amino acids such as arginine and cysteine that are essential for T cell proliferation and function (85). In addition, MDSCs produce ROS and NO, which disrupt TCR signaling pathways and induce T cell dysfunction and exhaustion.

Tregs further amplify the immunosuppressive landscape through multiple mechanisms. These include competing with effector T cells for co-stimulatory signals by engaging CTLA-4 on antigen-presenting cells, secreting IL-10 and TGF-β to directly suppress effector T cell activity, and consuming IL-2 to restrict the proliferative capacity of conventional T cells (97).

Together, TAMs, MDSCs, and Tregs form a highly coordinated immunosuppressive network. This multilayered inhibitory system ensures that even if a limited number of effector T cells manage to infiltrate the tumor parenchyma, they are rapidly rendered dysfunctional or exhausted, thereby severely limiting the efficacy of immunotherapeutic interventions in PDAC.

### 3.3 Metabolic competition and immune exhaustion

Metabolic competition represents a central barrier to effective antitumor immunity in PDAC, constituting a core mechanism of immune exclusion (98). Within the PDAC tumor microenvironment, tumor cells and immune cells engage in intense competition for metabolic substrates, establishing an energy-deprived niche that favors immune tolerance (99). PDAC tumor cells exhibit high glycolytic activity—even in the presence of oxygen—through a strongly activated Warburg effect, consuming vast amounts of glucose and thereby depriving infiltrating T cells of the essential energy required for their activation, proliferation, and effector functions (100).

Glucose depletion impairs the mammalian target of rapamycin (mTOR) signaling pathway in T cells, resulting in reduced proliferation, diminished cytotoxic activity, and early onset of functional exhaustion. In parallel, both tumor cells and immunosuppressive myeloid populations, such as MDSCs, highly express indoleamine 2,3-dioxygenase (IDO), which catalyzes the degradation of tryptophan into immunosuppressive metabolites like kynurenine (101). Kynurenine activates the aryl hydrocarbon receptor (AhR) pathway, promoting apoptosis of CD8^+^ T cells and the expansion of Tregs, further reinforcing an immunosuppressive TME (102).

In the context of elevated aerobic glycolysis, lactate accumulation within the TME further exacerbates immune dysfunction (103). Acidification of the local environment not only suppresses the cytotoxic function of CD8^+^ T cells and NK cells but also facilitates the polarization of macrophages toward the M2 immunosuppressive phenotype, thereby amplifying immune evasion mechanisms (104).

Moreover, dysregulated lipid metabolism plays a critical role in maintaining the suppressive function of TME-resident immune cells. Immunosuppressive cells such as Tregs, MDSCs, and TAMs upregulate lipid transporters and scavenger receptors—such as CD36 and fatty acid-binding proteins (FABPs)—to enhance fatty acid uptake and sustain their function under metabolic stress (105). In contrast, CD8^+^ T cells subjected to lipid peroxidation undergo oxidative stress–induced dysfunction, losing their cytotoxic potential and failing to sustain effective immune surveillance.

Collectively, this hostile metabolic landscape—characterized by glucose deprivation, amino acid catabolism, lactate accumulation, and aberrant lipid metabolism—drives progressive T cell exhaustion and establishes a metabolically repressive environment that severely limits the efficacy of immunotherapies in PDAC.

### 3.4 Checkpoint-independent mechanisms of immune tolerance

A crucial yet often overlooked dimension of immune evasion in PDAC extends beyond the well-characterized PD-1/PD-L1 checkpoint axis (106). Emerging evidence reveals that PDAC harnesses a network of alternative, checkpoint-independent immunosuppressive mechanisms to maintain its profoundly immune-resistant TME (106). These non-canonical pathways provide functional redundancy and compensation, helping to explain the consistently poor clinical response to ICB monotherapy in this disease.

One such mechanism involves the CD47/SIRPα axis. CD47 is commonly overexpressed on PDAC tumor cells and interacts with SIRPα on macrophages, transmitting a potent “don’t eat me” signal that inhibits phagocytosis and suppresses subsequent antigen presentation. This immune evasion tactic effectively dampens innate immune activation and limits downstream T cell priming. Another key pathway is the Galectin-9/TIM-3 axis, which is also upregulated in PDAC. TIM-3, expressed on dysfunctional T cells, NK cells, and myeloid populations, engages with Galectin-9 produced by tumor and stromal cells, leading to CD8^+^ T cell exhaustion or apoptosis and further impairing dendritic cell function and interferon-γ secretion (107).

In addition, the IDO–kynurenine–aryl hydrocarbon receptor pathway represents a metabolically integrated form of immunoregulation. Elevated IDO expression by tumor and antigen-presenting cells catalyzes the degradation of tryptophan into kynurenine, a metabolite that not only suppresses effector T cell function but also activates the AhR pathway, reinforcing the expansion and suppressive function of regulatory T cells (108).

Together, these PD-1/PD-L1-independent mechanisms form a complex and layered immunosuppressive architecture that allows PDAC to resist immunotherapeutic pressure. Their presence underscores the urgent need for rational combinatorial approaches that simultaneously target both canonical and non-canonical immune escape pathways—such as CD47, TIM-3, and IDO—in order to restore immune responsiveness in this therapeutically recalcitrant malignancy.

### 3.5 Clinical failures and mechanistic summary of immunotherapy resistance in PDAC

Clinically, numerous immunotherapeutic approaches targeting PDAC have failed to yield meaningful outcomes. Monotherapies using PD-1/PD-L1 inhibitors, as well as combinatorial strategies involving dual blockade of PD-1 and CTLA-4, have consistently shown disappointing results in PDAC patients (109). Major clinical trials—including KEYNOTE-158 and CheckMate 032—have reported objective response rates below 5% in microsatellite-stable (MSS) PDAC, which represents the vast majority of cases (110). Moreover, attempts to enhance antitumor immunity by co-administering myeloid-targeted agents, such as CSF1R inhibitors, have also failed to overcome the profound immunosuppression characteristic of the PDAC tumor microenvironment (111).

These clinical failures underscore the fact that immune resistance in PDAC is not driven by isolated activation of the PD-1/PD-L1 axis alone. Rather, it reflects a complex and multifactorial resistance program in which spatial barriers, immunosuppressive cell populations, metabolic constraints, and non-canonical immune checkpoints interact to sustain a highly suppressive ecosystem. This systemic model of immune evasion highlights the inadequacy of conventional checkpoint blockade strategies when applied to such an immunologically hostile tumor.

In summary, immune exclusion in PDAC should not be viewed as a standalone phenomenon, but as an emergent property of a highly interconnected and dynamic immunosuppressive landscape. The convergence of physical ECM barriers, a dense network of immunosuppressive cells, metabolic competition, and redundant immunoregulatory signaling pathways collectively constitutes the biological foundation underlying the universal failure of immunotherapy in PDAC. A comprehensive understanding of these mechanisms is not only essential for elucidating the root causes of therapeutic resistance, but also forms a critical theoretical framework for the development of next-generation combination immunotherapies—particularly those aimed at reprogramming the TME to restore effective antitumor immunity.

## 4 TME-targeting therapies currently applied in clinical cancer treatment

Given the intricate network of immune exclusion mechanisms delineated above, it has become increasingly clear that overcoming PDAC’s profound immunoresistance requires not only immune activation but also strategic reprogramming of its tumor microenvironment. In this context, modulation of the TME has emerged in recent years as a clinically relevant strategy to enhance the efficacy of cancer therapies. While many TME-directed agents remain under investigation, several therapeutic modalities have already entered clinical practice or are approved for specific malignancies. These clinically established strategies mainly target stromal remodeling, angiogenesis inhibition, immune checkpoint blockade, and CAF regulation. In PDAC, a cancer characterized by a highly desmoplastic and immunosuppressive TME, these approaches are often used in combination with chemotherapy to improve drug delivery and immune responsiveness.

Clinically validated TME-targeting therapies have become integral to cancer treatment paradigms. Immune checkpoint inhibitors (ICIs) such as pembrolizumab and nivolumab have demonstrated durable efficacy across multiple malignancies; however, their benefit in PDAC remains limited due to the highly immunosuppressive stroma. Anti-angiogenic therapies like bevacizumab and ramucirumab are established components of treatment for colorectal and lung cancers, and have shown potential to improve TME perfusion and immune infiltration in PDAC when combined with ICIs.

Additionally, chemotherapeutic agents, especially gemcitabine-based combinations, exert indirect TME-modifying effects by depleting stromal components and inducing immunogenic cell death. Losartan, an anti-fibrotic agent, and CSF1R inhibitors are being clinically repurposed to normalize the desmoplastic microenvironment and modulate tumor-associated macrophages, respectively, thereby enhancing immune responsiveness (**Table 1**).

**Table 1:** Clinically used TME-targeting therapeutic strategies in cancer, with emphasis on PDAC.

Therapeutic category	Representative agents	Mechanism of action	Approved/clinical use	Clinical relevance in PDAC	Ref.
Immune Checkpoint Inhibitors	Pembrolizumab, Nivolumab, Ipilimumab	Blockade of PD-1/PD-L1 or CTLA-4 pathways to restore T-cell activation	FDA-approved for multiple cancers (e.g., melanoma, NSCLC, MSI-high CRC)	Limited efficacy as monotherapy in PDAC; ongoing combination trials with chemotherapy or stromal modifiers	(112–114)
Anti-Angiogenic Therapy	Bevacizumab, Ramucirumab	Inhibit VEGF/VEGFR signaling to normalize tumor vasculature and improve immune infiltration	Approved for colorectal, lung, renal, and hepatocellular carcinoma	May enhance chemotherapy delivery and T-cell infiltration in PDAC; explored in combination with ICBs	(115, 116)
Stromal Remodeling Agents	Pegylated recombinant human hyaluronidase (PEGPH20)	Degrades hyaluronic acid to reduce stromal density and improve perfusion	Investigated in PDAC (Phase III HALO-301); not yet approved due to limited survival benefit	Demonstrated improved drug penetration; conceptually relevant for future TME modulation	(117)
Chemotherapy-Induced TME Modulation	Gemcitabine, Nab-paclitaxel, FOLFIRINOX	Indirectly remodel immune and stromal components; reduce tumor fibrosis	Standard-of-care for advanced PDAC	Induces immunogenic cell death and transiently reduces desmoplasia	(118)
Anti-Inflammatory and Myeloid-Targeting Agents	CCR2 inhibitor (PF-04136309), CSF1R inhibitor	Reduce tumor-associated macrophage recruitment and reprogram immunosuppressive myeloid cells	Evaluated in PDAC clinical trials (Phase I/II)	Improve T-cell infiltration and response to immunotherapy	(119, 120)
Anti-Fibrotic/CAF-Targeting Therapy	Losartan (angiotensin II receptor blocker)	Inhibits TGF-β–mediated fibrosis and normalizes extracellular matrix	Clinically used antihypertensive; repurposed in PDAC trials	Enhances drug delivery and immune access; used with FOLFIRINOX or ICBs	(121, 122)

Overall, the integration of TME-modulating therapies into established treatment regimens represents a clinically relevant approach for improving therapeutic outcomes, particularly in refractory malignancies such as PDAC.

## 5 Emerging technologies and novel therapeutic platforms for TME reprogramming in PDAC

Building upon the clinical advances and recognized limitations of current TME-targeting therapies, emerging technologies are now reshaping the landscape of PDAC treatment by enabling more precise and effective reprogramming of the tumor microenvironment. These innovations—spanning nanotechnology, single-cell and spatial multi-omics, synthetic biology, and artificial intelligence—are redefining how the PDAC microenvironment can be analyzed, targeted, and therapeutically manipulated.

### 5.1 Nanotechnology enables targeted delivery into PDAC’s dense stroma

The dense desmoplastic stroma of PDAC remains one of the most formidable obstacles to effective drug delivery and immune infiltration. Nanotechnology provides a versatile platform to overcome these barriers through both passive accumulation and active targeting strategies.

Passive targeting relies on the enhanced permeability and retention (EPR) effect, which enables nanoparticles (typically 50–150 nm) to preferentially accumulate within tumor tissues due to leaky vasculature and impaired lymphatic drainage (123). However, the highly fibrotic and poorly vascularized nature of PDAC limits EPR efficiency, necessitating the development of actively targeted nanocarriers.

Active targeting can be achieved by functionalizing nanoparticle surfaces with ligands, antibodies, or peptides that recognize specific components of the TME. For instance, hyaluronic acid (HA)-coated nanoparticles selectively bind to CD44-overexpressing CAFs, enabling targeted delivery of TGF-β inhibitors to disrupt stromal fibrosis and enhance T cell infiltration (124). Similarly, lipid nanoparticles encapsulating colony-stimulating factor 1 receptor (CSF1R) inhibitors or CD40 agonists have been shown to reprogram TAMs from an M2-like immunosuppressive phenotype toward a pro-inflammatory M1 state, thereby restoring antitumor immune activity (125).

Beyond biochemical targeting, stimuli-responsive nanoplatforms provide spatially and temporally controlled drug release in response to tumor-specific cues such as acidic pH, elevated ROS, or overexpressed matrix metalloproteinases (MMPs). For example, pH-sensitive nanocarriers can deliver cyclic dinucleotide (CDN) STING agonists specifically within the acidic PDAC microenvironment to activate dendritic cells and enhance cytotoxic T cell responses. Likewise, ROS-responsive nanozymes can neutralize oxidative stress, restore redox homeostasis, and improve T cell survival and function within the hostile TME (126).

A recent Nature Nanotechnology study demonstrated the potential of multifunctional nanoplatforms: a DNA-origami-based system was engineered to co-deliver a CXCR4 antagonist and anti–PD-1 antibody, effectively disrupting CAF-mediated immune exclusion and enhancing CD8^+^ T cell infiltration in murine PDAC models, leading to significant survival benefits (127).

Together, these advances illustrate that nanotechnology not only facilitates drug penetration through PDAC’s physical and immunological barriers but also enables precise modulation of stromal and immune interactions, making it a powerful adjunct to immunotherapy and targeted therapy in PDAC.

### 5.2 Single-cell and spatial multi-omics reveal high-resolution immune and stromal landscapes in PDAC

Single-cell and spatial multi-omics technologies have revolutionized our understanding of the PDAC tumor microenvironment by enabling high-resolution mapping of cellular heterogeneity, functional states, and spatial organization. Single-cell RNA sequencing has delineated distinct CAF subpopulations—myofibroblastic CAFs, inflammatory CAFs, and antigen-presenting CAFs—each contributing uniquely to immune evasion and stromal remodeling (29). Spatial transcriptomics further revealed that iCAFs preferentially localize near vasculature, secreting chemokines (e.g., CXCL12, IL-6) to form chemotactic “fences” that hinder effector T cell infiltration, while myCAFs cluster around tumor nests, producing ECM components that reinforce fibrotic encapsulation and restrict drug diffusion (128).

Beyond fibroblasts, integrated spatial multi-omics studies have uncovered the coordinated spatial arrangement of immunosuppressive cells. A landmark study in Cell demonstrated that TAMs, MDSCs, and exhausted CD8^+^ T cells co-localize within hypoxic and metabolically deprived niches, which correspond to sites of poor immune checkpoint blockade (ICB) efficacy (129). These findings highlight how metabolic and spatial cues converge to create localized “immune cold zones.”

Epigenetic profiling via scATAC-seq further complements transcriptomic data by uncovering chromatin-level determinants of T cell dysfunction. Exhausted CD8^+^ T cells exhibit closed chromatin states at lipid metabolism and mitochondrial biogenesis loci, constraining their metabolic adaptability. Interestingly, pharmacologic activation of AMPK or inhibition of HDAC3 has been shown to restore chromatin accessibility and reinvigorate effector functions (130).

Emerging single-cell metabolomics and spatial proteomics now provide an additional layer of insight, allowing researchers to map metabolic flux and cytokine gradients across the TME. Integrating these modalities offers a systems-level view of immune-stromal-metabolic crosstalk, paving the way for precision immunotherapy that targets specific suppressive niches or reprograms dysfunctional immune subsets.

### 5.3 Synthetic biology and engineered immune cells enable programmable immunomodulation

Synthetic biology introduces a powerful paradigm for programmable immune intervention in PDAC, enabling immune cells to sense, adapt, and remodel the TME in real time. Engineered immune cells—including macrophages, T cells, DCs, and Tregs—are now being designed as dynamic “living drugs” capable of integrating environmental cues into controlled therapeutic responses.

Chimeric antigen receptor macrophages (CAR-Ms) exemplify this approach. CD47-targeted CAR-Ms combine tumor phagocytosis with secretion of IL-12 and GM-CSF, leading to TAM repolarization and promoting CD8^+^ T cell infiltration (131). Advanced CAR-M designs integrate metabolic support circuits (e.g., IL-12–STAT4 or NF-κB–driven modules), maintaining macrophage activity even within the hypoxic, nutrient-depleted PDAC TME.

Logic-gated CAR-T cells enhance therapeutic precision through dual-input sensing of tumor-specific antigens (e.g., mesothelin, Claudin18.2) and suppressive signals (e.g., TGF-β, PD-L1) (132). These Boolean circuit-based constructs activate only under defined TME conditions, improving on-target efficacy while reducing systemic toxicity.

In parallel, CRISPR/Cas-based immune engineering enables immune cells to self-regulate cytokine secretion (e.g., STING agonists, IL-2, IL-15), providing localized immune amplification without inducing systemic cytokine storms (133). Additionally, CAR-DC platforms are being developed to strengthen antigen cross-presentation, while CAR-Treg depletion systems selectively eliminate immunosuppressive Tregs—together establishing a tunable immune balance in PDAC models (134).

Collectively, these synthetic biology innovations move beyond cytotoxic enhancement toward adaptive immune reprogramming, positioning engineered immune cells as next-generation precision therapeutics capable of navigating and reshaping PDAC’s immunosuppressive architecture.

### 5.4 Artificial intelligence catalyzes tme-targeted therapy design

Artificial intelligence (AI) serves as the integrative layer linking data, design, and therapeutic prediction across these platforms. By analyzing multidimensional datasets—including single-cell, spatial, metabolic, and histopathological information—AI models identify dominant immunosuppressive drivers and simulate rational intervention strategies (135).

For example, a DeepMind–Memorial Sloan Kettering model identified NOX4 as a key CAF effector and proposed dual inhibition of FAK and VEGFR2 to disrupt fibroblast–vascular crosstalk (129). AI-based combinatorial screening predicted that a triple regimen of STING agonist, LDHA inhibitor, and anti-PD-1 could overcome lactic-acid-driven MDSC accumulation and T-cell exhaustion (136).

Beyond drug design, generative algorithms now expedite the discovery of small-molecule immunomodulators, next-generation lipid nanoparticles, and nanobodies with optimized pharmacokinetic and immune-targeting profiles (137). Digital pathology platforms incorporating deep learning dynamically track TME remodeling, correlate spatial immune metrics with patient outcomes, and guide real-time therapeutic adjustment (138).

By transforming heterogeneous biological datasets into predictive and actionable models, AI closes the loop between mechanistic insight and clinical translation—accelerating the discovery, optimization, and personalization of TME-targeted therapies.

**Figure 4:**
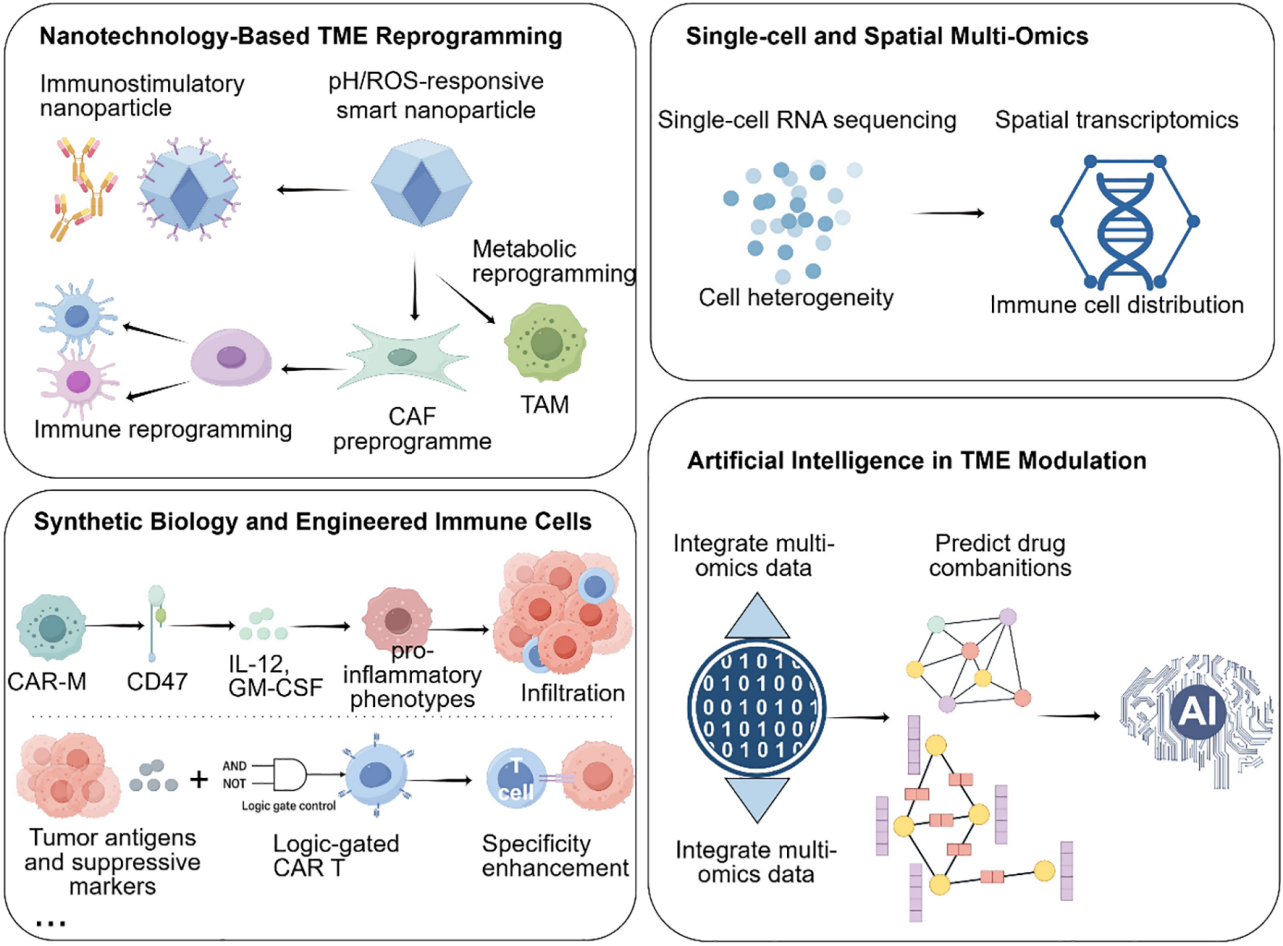
Emerging technologies for TME reprogramming in PDAC.

In summary, nanotechnology penetrates physical and biological barriers; multi-omics delivers spatially resolved insights; synthetic biology engineers precision immune effectors; and AI drives rational design and real-time optimization (**Figure 4**). Together, these innovations create a next-generation framework for mechanistic TME reprogramming in PDAC, forming a blueprint for future immunotherapeutic breakthroughs.

## 6 Clinical trials involving TME-directed combination immunotherapies

Recent clinical trials have explored the efficacy of TME-directed combination immunotherapies, aiming to enhance the antitumor immune response by targeting multiple components of the TME. These strategies typically combine immune checkpoint inhibitors (ICBs) with agents that modify the TME to overcome immunosuppressive barriers.

### 6.1 MORPHEUS-PDAC (atezolizumab + PEGPH20 vs. chemotherapy)

The MORPHEUS-PDAC Phase Ib/II trial evaluated the PD-L1 antibody atezolizumab combined with PEGPH20, a pegylated recombinant human hyaluronidase that degrades hyaluronic acid in the extracellular matrix (139). The combination achieved an objective response rate (ORR) of 8.3% and disease control rate (DCR) of 41.7%, compared with ORR of 5% in the chemotherapy control arm. While the treatment was well tolerated, no significant survival advantage was observed (median progression-free survival, 3.6 vs. 3.4 months), highlighting the difficulty of translating stromal remodeling into durable immune activation.

### 6.2 CXCR4 antagonist + PD-1 inhibitor (plerixafor/AMD3100 + cemiplimab)

A Phase II trial investigated the CXCR4 antagonist plerixafor (AMD3100) combined with the PD-1 antibody cemiplimab in metastatic PDAC patients (140). The dual blockade disrupted the CXCL12–CXCR4 axis, enhancing CD8^+^ T-cell infiltration. The regimen achieved stable disease in 36% of patients, with a median PFS of 2.8 months and OS of 6.9 months. Although no objective responses were observed, correlative analyses revealed reduced MDSC frequency and increased effector T-cell signatures, suggesting partial immunologic reprogramming.

### 6.3 PAAG regimen (penpulimab + anlotinib + chemotherapy)

In a multicenter Phase II study, the PD-1 inhibitor penpulimab was combined with the anti-angiogenic agent anlotinib and standard chemotherapy (nab-paclitaxel plus gemcitabine) as first-line therapy for metastatic PDAC (141). The triplet regimen achieved an ORR of 31.6%, DCR of 73.7%, and median PFS of 6.8 months, exceeding historical benchmarks for chemotherapy alone (ORR ≈ 23%, PFS ≈ 5.5 months). These results support the notion that TME normalization through vascular modulation enhances immune accessibility and checkpoint efficacy.

### 6.4 Anti-PD-L1 antibody + CSF1R inhibitor (durvalumab + pexidartinib)

An ongoing Phase I study (NCT02777710) is evaluating durvalumab (anti–PD-L1) in combination with pexidartinib (CSF1R inhibitor) in advanced pancreatic and colorectal cancers. Interim analyses indicate a manageable safety profile with partial responses in 2 of 25 PDAC patients (8%) and disease stabilization in 40%, accompanied by reductions in circulating M2-like TAMs and increased intratumoral CD8^+^ T-cell density. These findings provide early evidence that selective myeloid reprogramming can synergize with ICB to reshape the immunosuppressive milieu.

Collectively, these clinical trials underscore both the promise and the limitations of TME-directed combination immunotherapies in PDAC. While targeted modulation of stromal, vascular, and immune compartments has yielded modest improvements in clinical endpoints, durable responses remain elusive. The heterogeneous and dynamically evolving nature of the TME continues to constrain therapeutic efficacy, emphasizing that successful translation will depend on overcoming biological and technological barriers that limit immune reprogramming.

## 7 Challenges and future perspectives

Building upon the clinical findings summarized above, it has become increasingly evident that reprogramming the TME in PDAC remains a formidable challenge. Despite encouraging preclinical and early clinical signals, most TME-targeted strategies have yielded only modest and transient benefits in patients. This translational gap highlights the multifactorial resistance mechanisms that operate within the PDAC microenvironment—ranging from profound stromal desmoplasia and immune exclusion to metabolic and spatial heterogeneity—that collectively restrain durable antitumor immunity.

**Figure 5:**
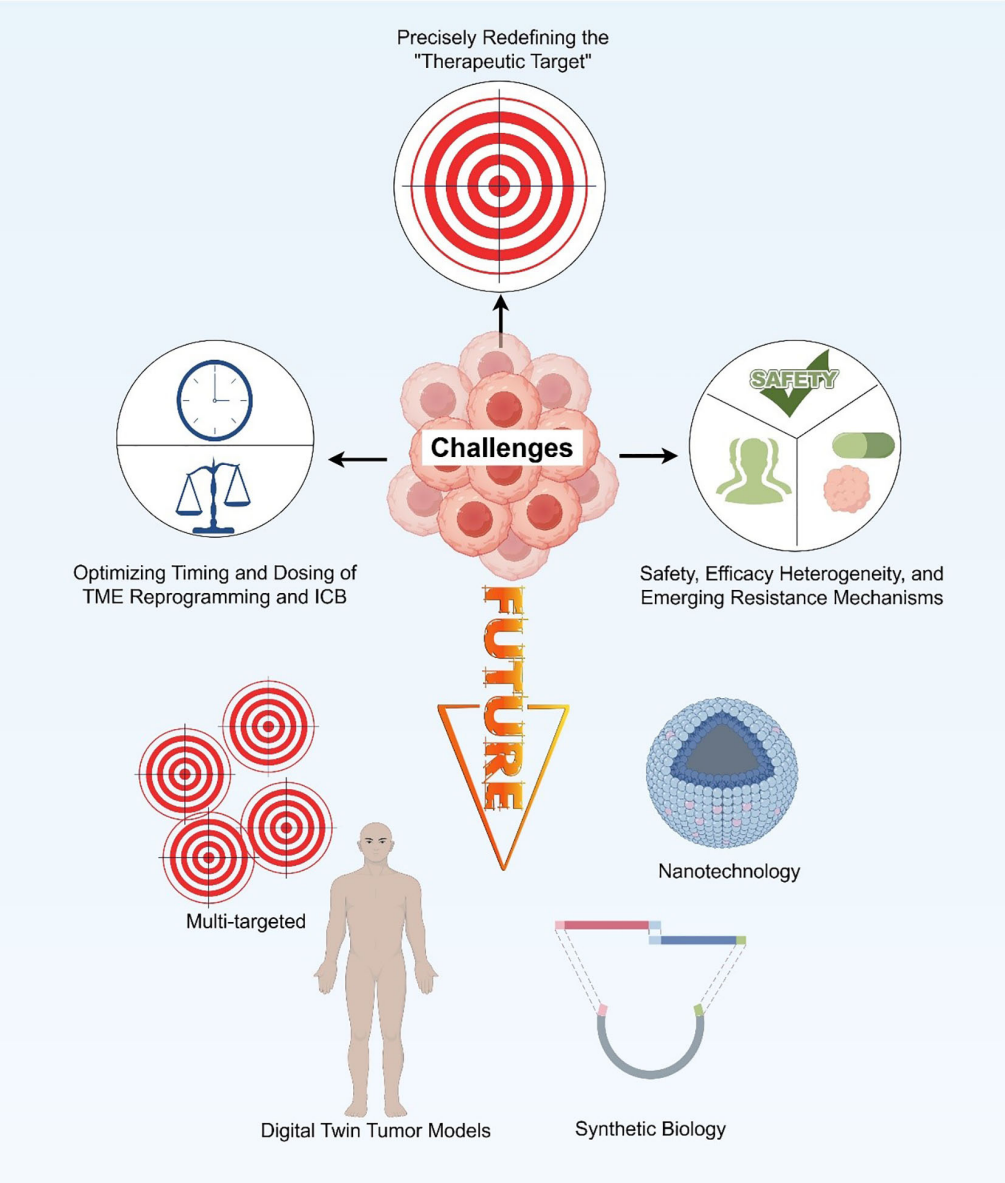
Challenges and future perspectives.

To achieve meaningful and lasting therapeutic responses, future research must move beyond single-target interventions toward an integrated understanding of the dynamic crosstalk among stromal, immune, and metabolic networks. Such progress will depend not only on refining the biological rationale for TME reprogramming but also on improving the technological platforms that enable precise modulation of the tumor milieu *in vivo*. In this context, addressing key challenges—including spatial and functional heterogeneity, therapeutic timing and sequencing, biomarker-driven patient stratification, and the rational design of multi-targeted personalized strategies—will be crucial to advancing the next generation of TME-directed immunotherapies (**Figure 5**).

### 7.1 Addressing spatial and functional heterogeneity of the TME: precisely redefining the “therapeutic target”

One of the most formidable barriers to precision immunotherapy in PDAC is the spatial and functional heterogeneity of the TME. Distinct subpopulations of CAFs, TAMs, MDSCs, and Tregs exhibit markedly different spatial distributions and immune-modulatory functions within the tumor mass. For instance, iCAFs often localize around vasculature and form chemokine-mediated exclusion zones, whereas myCAFs predominantly cluster around epithelial compartments to construct rigid ECM barriers (29). Similarly, metabolic gradients—including hypoxia and acidity—are spatially heterogeneous, directly shaping immune cell infiltration and effector functionality (142).

To effectively overcome the complex barriers posed by the PDAC tumor microenvironment, continued innovation in spatial multi-omics technologies is essential. Techniques such as spatial transcriptomics and proteomics are now making it possible to generate high-resolution, spatially resolved maps of immune architecture within tumors (143). When integrated with single-cell analyses, these tools can construct detailed “immuno-architectural atlases”, offering a new framework for understanding and targeting the immune landscape of PDAC. At the same time, progress in nanomedicine is enabling more precise therapeutic interventions. Advanced drug delivery systems—particularly nanoparticles engineered to respond to specific biochemical cues like hypoxia, reactive oxygen species, or acidic pH—can release their payloads in a site-selective manner, focusing treatment within the most immunosuppressive regions of the tumor. Further refinements, such as the incorporation of externally triggered release mechanisms (e.g., light, magnetic fields, or ultrasound), add another layer of spatial and temporal control, offering a promising strategy to address the heterogeneity of the PDAC microenvironment and improve therapeutic efficacy.

From a theoretical perspective, these efforts may converge into a novel conceptual framework termed “Spatial Immuno-Oncology” representing a next-generation subdiscipline that integrates spatial biology with immune modulation to inform the design of topologically tailored immunotherapies. As spatial resolution becomes an indispensable layer in understanding and treating cancer, this approach is poised to fundamentally reshape how we identify targets, deliver therapies, and measure responses in highly complex malignancies like PDAC.

### 7.2 Optimizing the timing and dosing of TME reprogramming and immune checkpoint blockade

A major translational bottleneck in integrating TME reprogramming with immune checkpoint blockade (ICB) lies in defining the temporal and quantitative parameters that govern synergistic efficacy. Conventional concurrent administration strategies often fail to achieve durable immune activation and may paradoxically heighten immune-related adverse events (144, 145). This limitation reflects the temporal heterogeneity and adaptive plasticity of the TME, in which immune, stromal, and metabolic compartments evolve continuously in response to therapeutic pressure.

During the course of therapy, TME components undergo dynamic state transitions that critically determine the success of subsequent immune interventions. For instance, TAMs can be epigenetically reprogrammed from an immunosuppressive M2-like phenotype toward a pro-inflammatory M1-like state, thereby enhancing antigen presentation and T cell recruitment (146). Likewise, cancer-associated fibroblasts display remarkable phenotypic elasticity, with shifts between myofibroblastic and inflammatory subtypes that modulate extracellular matrix stiffness, vascular permeability, and T cell infiltration (147). Simultaneously, restoration of immune metabolic homeostasis—such as normalization of glucose, tryptophan, or lactate flux—can recalibrate local nutrient competition and redox balance, enabling sustained effector T cell function within the metabolically hostile tumor milieu (148).

These dynamic and interdependent processes suggest that the immunologic readiness of the TME is a moving target, and that ICB efficacy depends on administering therapy at an optimal immune-activation window. Conceptually, this has prompted a “sequential or staged” immunotherapy paradigm, wherein TME-targeted reprogramming acts as a priming phase to dismantle immunosuppressive barriers before checkpoint blockade is introduced (149). For example, CSF1R inhibition or CCR2/CCL2 axis blockade may be used transiently to deplete or reprogram immunosuppressive myeloid subsets, generating a time-sensitive “window of opportunity” characterized by reduced myeloid-derived suppressor cell burden and enhanced antigen-presenting capacity. Within this permissive interval, PD-1/PD-L1 or CTLA-4 blockade can be administered to amplify T cell priming and effector expansion, maximizing the therapeutic impact while minimizing toxicity.

Moreover, metabolic preconditioning of the TME—through interventions such as lactate dehydrogenase A (LDHA) inhibition, arginase blockade, or IDO1 suppression—can function as a preparatory step that reverses T cell exhaustion, reestablishes mitochondrial fitness, and restores cytokine responsiveness, thereby sensitizing tumors to subsequent ICB exposure (150).

Looking ahead, the temporal optimization of combination immunotherapies is expected to move beyond empirical trial design toward data-driven personalization. Computational modeling, multi-omics time-course profiling, and AI-guided treatment simulation can be harnessed to predict individualized therapeutic trajectories—identifying when and how to modulate distinct TME compartments for maximal synergy. Such integrative strategies represent a conceptual shift from static combination therapy to adaptive, phase-specific immunomodulation, embodying the next frontier in precision immuno-oncology.

### 7.3 Patient heterogeneity and personalized immunotherapy in PDAC

One of the major obstacles to effective immunotherapy in PDAC is the pronounced heterogeneity among patients at genomic, epigenetic, and microbial levels. Unlike highly immunogenic tumors such as melanoma or lung cancer, PDAC typically exhibits a low tumor mutational burden (TMB), resulting in fewer neoantigens and limited immune recognition (151). However, a small subset of PDACs (~1–2%) harbor microsatellite instability–high (MSI-H) or deficient mismatch repair (dMMR) phenotypes, which are associated with increased TMB, higher PD-L1 expression, and greater responsiveness to immune checkpoint blockade (152). Clinical trials have demonstrated durable responses to pembrolizumab in MSI-H/dMMR PDAC, leading to FDA approval of PD-1 blockade for this molecularly defined subgroup (153).

Beyond genomic variability, the tumor microbiome has emerged as a critical determinant of immunotherapy response. Distinct microbial communities within PDAC can modulate immune infiltration and drug metabolism. For instance, *Gammaproteobacteria* have been shown to metabolize gemcitabine into inactive forms, contributing to chemoresistance (154). More importantly, specific commensal bacteria such as *Bifidobacterium* and *Akkermansia muciniphila* can enhance antitumor immunity by promoting dendritic cell activation and improving the efficacy of PD-1 blockade (155, 156). Conversely, dysbiotic microbial signatures rich in *Fusobacterium nucleatum* or *Enterobacteriaceae* are linked to immunosuppressive myeloid infiltration and poor clinical outcomes (157).

Furthermore, epigenetic and metabolic heterogeneity within the TME introduces additional complexity. For example, differential activation of KRAS downstream pathways or variations in CAF-derived cytokine profiles can reshape local immune composition, resulting in variable sensitivity to immunomodulatory agents (29, 158). Integrating multi-omics profiling—including genomics, metabolomics, and microbiome sequencing—will be essential for identifying patient-specific immunological landscapes and optimizing therapeutic combinations. Ultimately, understanding and stratifying PDAC patients based on these heterogeneity factors may enable precision immunotherapy and improve clinical response rates.

### 7.4 Core challenges in clinical translation

Despite the growing promise of TME reprogramming strategies in enhancing antitumor immunity across various cancer models, their clinical translation remains hampered by three major barriers—safety, efficacy heterogeneity, and emerging resistance mechanisms—each necessitating deeper mechanistic understanding and refined therapeutic design.

First, safety concerns remain paramount. TME modulation often involves targeting multiple signaling axes in parallel, particularly when combined with immune checkpoint blockade (91). Such multifaceted interventions can provoke systemic immune activation, resulting in severe immune-related adverse events such as cytokine release syndrome (CRS), autoimmune organ injury, and hematologic or hepatic toxicity. Of particular concern is the risk of off-target immunotoxicity, where broad myeloid or stromal reprogramming may inadvertently perturb normal tissue immune homeostasis. For example, inhibition of CSF1R or CCR2 pathways can disrupt macrophage balance, leading to excessive inflammatory responses and systemic toxicity (159).

Moreover, excessive stromal depletion represents a key controversy in TME-targeted therapy. Although degradation of the extracellular matrix or depletion of cancer-associated fibroblasts can enhance immune cell infiltration and drug delivery, preclinical evidence indicates that over-disruption of stromal architecture may paradoxically promote tumor invasion and metastasis (160). Stromal components such as αSMA^+^ fibroblasts and collagen networks not only restrict tumor spread but also maintain vascular integrity and mechanical containment. Their loss may lead to vascular leakiness, hypoxia, and epithelial–mesenchymal transition (EMT), ultimately accelerating tumor progression. These risks underscore the need for selective and reversible stromal reprogramming rather than complete ablation.

Second, pronounced inter-patient and intra-tumoral heterogeneity in therapeutic response remains a substantial challenge. Even under standardized treatment regimens, patient responses can vary dramatically—not only due to intrinsic genomic differences but also as a result of spatiotemporal dynamics within the TME. Currently, the lack of reliable, real-time biomarkers capable of capturing TME remodeling constrains the clinician’s ability to adjust therapy based on mechanistic feedback, leaving treatment decisions heavily reliant on empirical judgment rather than data-driven precision. This severely limits the implementation of truly personalized TME-based strategies.

Third, the emergence of acquired resistance mechanisms is becoming a new frontier in the field. The plasticity of the TME—especially in immunologically “cold” tumors such as PDAC—enables rapid adaptation in response to targeted interventions (81). When a dominant immunosuppressive axis (e.g., CSF1/CSF1R) is effectively inhibited, compensatory immune escape pathways such as Galectin-9/TIM-3 or CD47/SIRPα are frequently upregulated (161). Moreover, metabolic rewiring—such as a shift from lactate fermentation to fatty acid oxidation—allows both tumor and stromal cells to circumvent metabolic blockade while sustaining an immunosuppressive milieu (162).

Finally, therapeutic balance is essential. Tumor adaptation and plasticity may enable malignant cells to restore immune suppression over time, while uncoordinated or excessive immune activation during TME reprogramming can lead to unintended systemic or local immune perturbations (163). Maintaining stromal integrity and immune equilibrium therefore represents a central challenge—requiring precision delivery systems, temporal control of therapeutic dosing, and integration of multi-omic biomarkers to guide dynamic adjustment. Collectively, these considerations highlight that successful clinical translation of TME-targeted strategies will depend not only on overcoming immunosuppressive barriers but also on preserving the homeostatic balance of the tumor–stroma–immune ecosystem.

### 7.5 Future directions: toward multi-target synergy and personalized TME remodeling

Addressing these challenges requires a balanced, adaptive approach. Integrating real-time biomarkers, spatial multi-omics, and computational modeling can guide dynamic modulation of therapeutic intensity, maximizing durable antitumor immunity while minimizing adverse effects. The convergence of TME-targeted interventions with AI-assisted diagnostics, digital twin modeling, and programmable therapeutic platforms is expected to enable context-aware, patient-specific immunological reprogramming, extending beyond PDAC to other immunologically “cold” malignancies such as prostate cancer and glioblastoma.

Digital Twin Tumor models are poised to transform precision medicine in PDAC. By integrating spatial transcriptomics, single-cell RNA sequencing, proteomics, metabolomics, and longitudinal imaging, these models reconstruct dynamic, patient-specific representations of the TME (164). Advanced computational frameworks—including graph neural networks, multi-omics integration algorithms, and reinforcement learning—allow simulation of tumor evolution, capture of cellular interactions, and iterative updating of model parameters based on patient-derived data. Digital twins can thus predict therapeutic outcomes under different interventions, supporting rational design of personalized drug combinations, dosing regimens, and treatment sequences.

Synthetic biology and nanotechnology further expand TME-targeted strategies. Next-generation CAR-T cells engineered with environmental sensing modules can dynamically modulate cytotoxic programs in response to key TME cues such as TGF-β, lactate, and ROS (165, 166). Meanwhile, AI-guided nanorobots may enable spatially precise delivery of immunostimulatory agents, metabolic inhibitors, or gene-editing tools within specific TME regions, enhancing both specificity and therapeutic efficacy.

Ultimately, these approaches converge toward an adaptive, context-aware immunological intervention paradigm, shifting from standardized treatments to highly responsive, patient-tailored therapies. Such an integrated strategy holds transformative potential for PDAC and other immunologically “cold” tumors, establishing a framework for precision TME remodeling in future cancer therapy.

## 8 Conclusion

Future therapeutic strategies will need to move away from simplistic, single-target approaches toward more dynamic, multidimensional interventions that tackle the complex layers of immune suppression within the TME. This requires not only the modulation of immune cells, such as CAFs, TAMs, and MDSCs, but also the correction of metabolic imbalances, remodeling of the vasculature, and targeted delivery of therapies to specific TME regions. The advent of technologies such as spatial transcriptomics, nanomedicine, and AI-driven models will provide unprecedented opportunities for real-time monitoring and personalized treatment strategies. Ultimately, TME reprogramming represents a shift in how we conceptualize PDAC, viewing it not just as a malignant epithelial disease, but as a product of persistent immune dysregulation. As such, TME remodeling has the potential to become the cornerstone of effective PDAC therapy, ultimately overcoming the long-held perception of PDAC as an immunologically “untreatable” cancer.
